# Four Food and Drug Administration draft guidance documents and the REGROW Act: A litmus test for future changes in human cell‐ and tissue‐based products regulatory policy in the United States?

**DOI:** 10.1002/term.2683

**Published:** 2018-05-21

**Authors:** Kazuo Yano, Alessondra T. Speidel, Masayuki Yamato

**Affiliations:** ^1^ Institute of Advanced Biomedical Engineering and Science Tokyo Women's Medical University Tokyo Japan; ^2^ Research Institute for Science and Engineering Waseda University Tokyo Japan; ^3^ Cooperative Major in Advanced Biomedical Sciences Joint Graduate School of Tokyo Women's Medical University and Waseda University Tokyo Japan

**Keywords:** accelerated approval, conditional and time‐limited authorization, conditional market authorization, Food and Drug Administration (FDA), guidance, human cells, tissues, and cellular and tissue‐based products (HCT/Ps), market authorization under exceptional circumstance

## Abstract

Modern regenerative medicine research has expanded well past the development of traditional drugs and medical devices with many promising new therapies encompassing an increasingly diverse range of substances, notably cell‐based therapies. These substantial recent developments and the progress in the health care and therapeutics fields necessitate a new regulatory framework agile enough to accommodate these unique therapies and acknowledge their differences with traditional pharmaceuticals. In the United States, recent proposed changes in the regulatory framework for autologous human cells, tissues, and cellular and tissue‐based products (HCT/Ps) and their perceived risk–benefit analysis for patients remain controversial in the scientific field. To provide perspective on of the current status of the most recent attempts to redefine and conceptualize these changes in the United States, we will examine 4 draft guidance documents implemented by the Food and Drug Administration in interpreting relevant concepts and terminology pertaining to HCT/Ps: the Bipartisan Policy Center think tank report, “Advancing Regenerative Cellular Therapy: Medical Innovation for Healthier Americans,” the proposed REGROW Act for HCT/Ps, and the current 24 Food and Drug Administration‐approved HCT/Ps and related products in the United States.

## 1 INTRODUCTION

The nascent, exponentially growing regenerative medicine field has introduced new classes of therapies and treatments that have outgrown the existing legislative parameters internationally. In order to efficiently deliver these new therapies to patients in need, countries have been seeking amendments to their existing legislation. Of particular interest within these classes of treatments are stem cell and other regenerative cell‐based therapies, which have gained popularity and garnered excitement even amongst the general public over the past several decades.

In the United States, original attempts by the Food and Drug Administration (FDA) to address cell therapy regulations were based on modification of existing policies for chemical drugs, biologics, and vaccines. The relevant regulatory framework is included in Sections 351 and 361 of the Public Health Service (PHS) Act of 1944, which define biological products and provide the FDA with the authority to prevent the spread of communicable diseases, respectively (FDA, 2017k), and in Title 21 of the Federal Code of Federal Regulations of 2001, Part 1271 (referred to as 21 CFR Part 1271), which outlines regulations for human cells, tissues, and cellular and tissue‐based products (HCT/Ps; FDA, 2017d). The FDA defines HCT/Ps as any product “containing or consisting of human cells or tissues intended for implantation, transplantation, infusion, or transfer into a human recipient” (FDA, 2017d). In 2001, HCT/P therapies were broken down into the following three‐tier regulation classification system (FDA, 2001c):

– First tier (low risk): Considered current medical practice and not subject to FDA preapproval, that is, organ transplant, blood transfusion as defined 21 CFR Part 1270 (FDA, 2017c) or 21 CFR Part 1271.15 (FDA, 2017d).
– Second tier (middle risk): Also referred to as “361 HCT/P products” after Section 361 of the PHS Act, which governs their use. Eligible products (a) must be minimally manipulated for (b) homologous use (as determined by advertising and labelling of product), (c) cannot be combined with “another article, except for water, crystalloids, or a sterilizing, preserving, or storage agent” (these exemptions must not present clinical safety concerns), and (d) do “not have a systemic effect” and are “not dependent upon the metabolic activity of living cells for their primary function” (unless they are for autologous use, for allogeneic use in the first‐degree or second‐degree blood relative, or for reproductive use; FDA, 2017d). Such 361 HCT/Ps are not subject to premarket FDA approval or clearance (21 CFR Part 1271.10; FDA, 2017d).
– Third tier (high risk): Also referred to as “351 products” after Section 351 of the PHS Act that governs their regulation. These include any cell‐based therapies that do not fulfil all four second‐tier criteria and therefore require a full premarket biologics license application (BLA) and must follow the same premarket and postmarket regulation as medical devices, drugs, or biologics (21 CFR Part 1271.20; FDA, 2017d). Thus, not all 351 products are HCT/Ps, but some HCT/Ps are regulated as 351 products.

Most would consider this patchwork system to be inefficient in accommodating the inherent differences between traditional pharmaceuticals and cell‐based products. However, between 2001 and the enactment of the 21st Century Cures Act, the FDA made minimal changes to these regulations, with most changes even further limiting the cell types eligible for the pathways available within the existing lower risk tiers.

As a result of the slow‐to‐change FDA, patients turned both to stem cell tourism, pursuing cell therapy treatments in countries with less regulation, and to the, as of 2016, 570 domestic clinics selling unapproved stem cell therapies that have sprouted up across the United States (Turner & Knoepfler, 2016). Recently, there has been much media attention given to the deregulation of stem cell therapies in Texas through a recent law that allows for patients to access unapproved stem cell products if a physician approves and oversees the treatment (Servick, 2017). In early August 2017, the U.S. Senate unanimously voted to pass a “Right to Try” bill, which gives terminally ill patients access to experimental therapies that have not yet been approved by the FDA (Pear & Kaplanug, 2017). This bill that has been part of a lager movement that has resulted in the rapid adoption of similar “Right to Try” legislation by 38 of the 50 U.S. states as of October 2017 (Goldwater Institute, 2017). Although this bill still requires ratification by Congress, the current political climate in the United States appears to be ripe for a bill supporting further deregulation of the federal government to pass. If passed, this bill could be the first to restrict the FDA's current control over the regulation of cell therapies (FDA, 2017d).

Given this political context, this article will focus on providing an overview of the regulatory momentum that has been gathering over the past 5 years in the United States to specifically reframe the translation policies for HCT/Ps. This review will outline the four draft guidance documents released by the FDA before the enactment of the 21st Century Cures Act and the accompanying regenerative medicine advanced therapy (RMAT) draft guidance documents; a recent report with recommendations on cell therapy policy released by a U.S. think tank known as the Bipartisan Policy Center (BPC); and the Reliable and Effective Growth for Regenerative Health Options that Improve Wellness Act of 2016 (REGROW Act), a body of legislation proposed during the previous session of the U.S. Congress. These documents provide a useful context for more deeply understanding the forces at work shaping the current and possibly future regulatory policies on HCT/P in the United States.

## 2 FOUR DRAFT GUIDANCE DOCUMENTS FRAMING FDA PERSPECTIVES ON HCT/Ps

Draft guidance documents outline the FDA's current definition and recommendations on certain topics within their jurisdiction. They are not official regulations unless they are officially adopted, but they provide useful insight into the FDA's perspective on certain controversial topics and can serve as a litmus test for organizations applying for certain FDA‐warranted approvals. The FDA usually disseminates such documents in part to provide advice and recommendations to the relevant establishments and organizations that may need to apply for product approval, but also to collect feedback and commentary from the pertinent stakeholders on the topics before finalizing the document. The four criteria necessary for products to qualify for the second tier within the FDA's HCT/P regulatory hierarchy have been the focus of much of the regulatory debate related to cell therapies. In particular, most of the recent debate has been centred around clarifying the FDA's definitions of “homologous use” and “minimal manipulation,” two requirements relevant to “the 361 HCT/Ps” that fall under this second tier and are exempt from FDA premarketing regulation. Between 2014 and 2015, four controversial FDA draft guidance documents were released, further narrowing the Administration's definitions of these terms:

1. Same Surgical Procedure Exception Under § 1271.15(b): Questions and Answers Regarding the Scope of the Exception (October 2014; FDA, 2014c)
2. Minimal Manipulation of Human Cells, Tissues, and Cellular and Tissue‐Based Products (December 2014; FDA, 2014b)
3. Human Cells, Tissues, and Cellular and Tissue‐Based Products (HCT/Ps) from Adipose Tissue (December 2014; FDA, 2014a)
4. Homologous Use of Human Cells, Tissues, and Cellular and Tissue‐Based Products (October 2015; FDA, 2015a)

As part of the intent behind draft guidance documents is to collect feedback from the relevant stakeholders before finalization, and because the content of these draft guidance documents was controversially narrowing definitions of some FDA preapproval exclusion criteria, the FDA was urged to hold public hearings to discuss the draft guidance documents' content. Due to considerable public interest, the original hearing was converted to a 2‐day public hearing and postponed from April 13, 2016, to September 12 and 13, 2016, to allow stakeholders additional time to prepare (FDA, 2016c, 2016d). A total of 76 speakers, including patients and stakeholders from industry, academia, and private consultancies, participated in the public hearing (FDA, 2016g, 2016e).

Table 1 outlines the titles of the current four FDA draft guidance documents pertaining to cell therapy‐related topics, their main points for consideration, and the reference information in the Federal Register. In the subsequent subsections, we expand on the key details within these draft guidance documents.

**Table 1:** Four recently issued draft guidance documents on human cells, tissues, and cellular and tissue‐based products from the Food and Drug Administration

Title of guidance	Purpose	Reference
Same Surgical Procedure Exception Under § 1271.15(b): Questions and Answers Regarding the Scope of the Exception; Draft Guidance for Industry (Same Surgical Procedure Exception Draft Guidance)	Provides answers to common questions regarding the scope of the same surgical procedure exception	Federal Register, October 23, 2014 (79 FR 63348)
Same Surgical Procedure Exception: Questions and Answers Regarding the Scope of the Exception; Guidance for Industry; Availability (Same Surgical Procedure Exception and Adipose Tissue Final Guidance)	Provides tissue establishments and health care professionals with FDA's current thinking on the scope of an exception set forth in human cells, tissues, and cellular and tissue‐based products (HCT/Ps) regulation	Federal Register, November 17, 2017 (82 FR 54289)
Minimal Manipulation of Human Cells, Tissues, and Cellular and Tissue‐Based Products; Draft Guidance for Industry and Food and Drug Administration Staff (Minimal Manipulation Draft Guidance)	Provides recommendations for meeting the criterion of minimal manipulation	Federal Register, December 23, 2014 (79 FR 77012)
Regulatory Considerations for Human Cells, Tissues, and Cellular and Tissue‐Based Products: Minimal Manipulation and Homologous Use; Guidance for Industry and Food and Drug Administration Staff; Availability (Minimal Manipulation, Homologous Use, and Adipose Tissue Final Guidance)	Provides HCT/Ps manufactures, health care providers, and FDA staff with FDA's current thinking on the regulatory criteria of minimal manipulation and homologous use	Federal Register, November 17, 2017 (82 FR 54290)
Human Cells, Tissues, and Cellular and Tissue‐Based Products (HCT/Ps) from Adipose Tissue: Regulatory Considerations; Draft Guidance for Industry (Adipose Tissue Draft Guidance)	Provides those who manufacture and use adipose tissue with recommendations for complying with regulatory framework for HCT/Ps	Federal Register, December 24, 2014 (79 FR 77414)
	FDA does not intend to finalize the Adipose Tissue Draft Guidance, which is now withdrawn	
Same Surgical Procedure Exception: Questions and Answers Regarding the Scope of the Exception; Guidance for Industry; Availability (Same Surgical Procedure Exception and Adipose Tissue Final Guidance)	Provides tissue establishments and health care professionals with FDA's current thinking on the scope of an exception set forth in HCT/Ps regulation	Federal Register, November 17, 2017 (82 FR 54289)
	This guidance supersedes the Adipose Tissue Draft Guidance	
Homologous Use of Human Cells, Tissues, and Cellular and Tissue‐Based Products; Draft Guidance for Industry and Food and Drug Administration Staff (Homologous Use Draft Guidance)	Provides recommendations for interpreting the homologous use criterion	Federal Register, October 30, 2015 (80 FR 66850)
Regulatory Considerations for Human Cells, Tissues, and Cellular and Tissue‐Based Products: Minimal Manipulation and Homologous Use; Guidance for Industry and Food and Drug Administration Staff; Availability (Minimal Manipulation, Homologous Use, and Adipose Tissue Final Guidance)	Provides HCT/Ps manufactures, health care providers, and FDA staff with FDA's current thinking on the regulatory criteria of minimal manipulation and homologous use	Federal Register, November 17, 2017 (82 FR 54290)

*Note*. Draft Guidance Relating to the Regulation of Human Cells, Tissues, or Cellular or Tissue‐Based Products; Public Hearing; Request for Comments (Federal Register, 80(210): 66845–66847, Friday, October 30, 2015): The public hearing was announced to be held on April 13, 2016, from 8:00 a.m. to 5:00 p.m. but was postponed to September 12 and 13 from 8:00 a.m. to 5:00 p.m.

Draft Guidance Relating to the Regulation of Human Cells, Tissues, and Cellular and Tissue‐Based Products; Rescheduling of Public Hearing; Request for Comments (Federal Register, 81(78), 23661–23664, Friday, April 22, 2016): The public hearing was announced to be held on September 12 and 13, 2016, from 8:00 a.m. to 5:00 p.m.

Draft Guidance Relating to the Regulation of Human Cells, Tissues, and Cellular and Tissue‐Based Products; Extension of Comment Periods (Federal Register, 81(78), 23664–23666, Friday, April 22, 2016): Due date for submitting either electronic or written comments was extended by September 27, 2016.

Part 15 Hearing: Draft Guidance Relating to the Regulation of Human Cells, Tissues, or Cellular or Tissue‐Based Products. The hearing was held on September 12 and 13, and hearing minutes were published on http://www.fda.gov/downloads/biologicsbloodvaccines/newsevents/workshopsmeetingsconferences/ucm532350.pdf.

### 2.1 Same Surgical Procedure Exception Under § 1271.15(b): Questions and Answers Regarding the Scope of the Exception (FDA, 2014c)

In this draft guidance, the FDA narrowly defines the “same surgical procedure exception” for HCT/P uses that would fall outside of FDA regulation. In order for the surgical exception to apply, three criteria must be met: (a) HCT/Ps must be removed from and implanted into the same patient, (b) the surgical removal and implantation must occur within the same procedure, and (c) HCT/Ps must remain “in their original form,” so that the “communicable disease risks” would remain the same as those usually associated with surgery (FDA, 2014c). Eligible procedures were narrowly defined to involve HCT/P removal and implantation back into the same patient within a single operation performed at the same establishment. Rinsing or cleaning, labelling, and temporary storage of HCT/P were the only cell processing steps allowed within what could be considered the same surgical procedure exception.

“Autologous use” is the term given to describe the first criteria for surgical exception and is further defined in this draft guidance to include “the implantation, transplantation, infusion, or transfer of human cells or tissue back into the individual from whom these cells or tissue were originally removed” (FDA, 2014c). Although this guidance presents additional criteria for exemption from FDA preapproval in addition to those outlined in the first and second tiers, interestingly, this guidance limits eligible procedures to include only those that occur within the same patient, whereas the earlier 361 product criteria viewed autologous use to be the same level of risk as allogeneic use in a close relative. This guidance was finalized with the same title in November 2017 (FDA, 2017n).

### 2.2 Minimal Manipulation of Human Cells, Tissues, and Cellular and Tissue‐Based Products (FDA, 2014b)

In this draft guidance, the FDA provide further definition of minimal manipulation expanding on the original description given in 21 CFR Part 1271 and providing numerous examples of procedures that qualify as minimal manipulation or would otherwise be considered more than minimal manipulation (FDA, 2014b). As in the original regulation, the definition for minimal manipulation is divided into two sections within this guidance depending on whether the relevant cells/tissues are classified as structural tissues or are considered cells/nonstructural tissues. Structural tissues are defined as those that serve as a barrier or provide support within the body, such as bone, skin, blood vessels, or adipose tissue. Nonstructural tissues are defined to “serve predominantly metabolic or other biochemical roles in the body, such as hematopoietic, immune, and endocrine functions” and include tissues such as cord blood, bone marrow aspirate, lymph nodes, and pancreatic tissue (FDA, 2014b).

Minimal manipulation for structural tissues was originally defined as processing that does not alter the “original relevant characteristics of the tissue, relating to the tissue's utility for reconstruction, repair, or replacement” (FDA, 2017d). Such relevant characteristics are further defined in this draft guidance to include measures of structural integrity such as flexibility, strength, and compressibility. Changing the size or shape of structural tissues, such as shaping a bone graft, is generally considered minimal manipulation unless the processing methods change the physical composition of tissues in a manner that impacts their relevant functionality. Grinding, lyophilizing, and packing an amniotic membrane as powder, however, would be considered more than minimal manipulation because these alter the membrane's physical integrity, impacting its relevant function as a membranous barrier. Separation of structural tissue into components or isolation of cells can be considered minimal manipulation if the structural tissue's original characteristics and function are preserved. For example, the removal of the epidermis and some connective tissue from a skin sample in preparation of a decellularized dermal graft would qualify as minimally manipulated as the resulting graft still retains the relevant barrier function of skin. However, the chemical manipulation of collagen cross‐linking in ligament tissue would be considered more than minimal manipulation as the interference with the structure of the collagen impacts the strength and structural integrity of the ligament.

For cells or nonstructural tissues, the original regulation considers minimal manipulation “as processing that does not alter the relevant biological characteristics of cells or tissues” (FDA, 2017d). This guidance defines the “biological characteristics” that should be preserved in the minimal manipulation of cells and nonstructural tissue to include metabolic activity, differentiation state, and proliferation capacity (FDA, 2014b). Extraction and concentration of haematopoietic stem/progenitor cells from peripheral blood for transplantation would generally be considered minimal manipulation procedures because the concentrated stem/progenitor cells would retain their relevant ability to repopulate the bone marrow. However, the processes of selection and tailored culture of large numbers of cells from placental/umbilical cord blood for repopulating bone marrow would be more than minimal manipulation as these processes would alter the cells' relevant capacity for self‐renewal and multipotency.

This draft guidance was finalized in November 2017 in a guidance document entitled “Regulatory Consideration for Human Cells, Tissues, and Cellular and Tissue‐Based Products: Minimal Manipulation and Homologous Use” (FDA, 2017m). Compared with the draft guidance, this final guidance provides further discussion and more detailed clarification on whether procedures such as decellularization of structural tissues and cryopreservation storage would qualify as minimal manipulation or that would be considered more than minimal manipulation (FDA, 2017n). Further clarification, however, would be useful in qualifying whether or when certain specific procedures, such as fluorescence‐activated cell sorting, centrifugation, or treatment with antibiotics, qualify as minimal manipulation.

### 2.3 Human Cells, Tissues, and Cellular and Tissue‐Based Products (HCT/Ps) from Adipose Tissue: Regulatory Considerations (FDA, 2014a)

In recent years, numerous unlicensed clinics in the United States have sprouted up selling unapproved adipose‐derived stem cell products for treatment of a variety of diseases and conditions. The popularity of adipose‐derived stem cell products derives from their easy and reasonably safe harvest from liposuction procedures as well as the vague and imprecise description of the treatments implementing these cells as “experimental,” which attracts desperate patients hoping to be on the cutting edge of a cure (Taylor‐Weiner & Graff Zivin, 2015). In an attempt to clarify regulatory recommendations for cells derived from this adipose tissue, the FDA released a draft guidance specific to HCT/Ps derived from this tissue. The draft guidance begins by breaking down the four major criteria that qualifying HCT/Ps must fulfil to fit within the second tier of the HCT/P regulatory hierarchy and provides examples of how these criteria relate to adipose tissue in particular. The draft guidance also includes details on how HCT/Ps that do not meet all four criteria are regulated and outlines the procedures that manufacturers must adhere to in their dissemination. The draft guidance only provides significant interpretations and/or examples of the first and second criteria applied to adipose tissue‐derived HCT/Ps. Interpretations of the third and fourth criteria essentially repeated the existing legislation without providing further exceptions or significant further clarifications and, therefore, were skipped in our summary.

In consideration of the first criteria, minimal manipulation status of adipose tissue‐derived HCT/Ps, the FDA typically classifies adipose tissue as a structural tissue as it functions to “cushion and support other tissues in the subcutaneous layer and skin” (FDA, 2014a). Processes that qualify as minimal manipulation of adipose tissue include “aliquoting, rinsing, removal of macroscopic debris, and freezing” (FDA, 2014a). Processing that decellularizes, isolates certain essential cellular components, or otherwise decomposes adipose tissue in a manner that it cannot perform its relevant functions of “cushioning and support” are considered more than minimal manipulation (FDA, 2014a).

Interpretation of the second criteria, homologous use of adipose tissue‐derived HCT/Ps, requires that the product perform a relevant adipose tissue function in the recipient. HCT/Ps from adipose tissue replacing an adipose tissue defect, such as in cosmetically filling spaces in a patient's face or hands, would be considered homologous use. However, use of HCT/Ps from adipose tissue to treat bone and joint disease would generally be considered nonhomologous uses, as adipose tissue does not have bone‐, or joint‐specific functionality.

The draft guidance included a section outlining the application of adipose tissue‐derived HCT/Ps under the exemptions of the “same surgical procedure” (FDA, 2014a). In order for HCT/Ps from adipose tissue to qualify for the exemptions under the same surgical procedure, the adipose tissue could only be “rinsed, cleansed, or sized” in between removal and autologous implantation (FDA, 2014a). Processing steps including “cell isolation, cell expansion, or enzymatic digestion” would disqualify the HCT/Ps from this exemption (FDA, 2014a). For example, lipoaspirate that is centrifuged to remove blood and extracellular fluid and washed in sterile saline solution before injection back into the same patient's subcutaneous space would qualify as the same surgical procedure. However, if stem cells are removed from the lipoaspirate before injection back into the same patient, this would be considered a manipulation that would alter the cushioning function of the adipose tissue and therefore would not qualify under the same surgical procedure exemption.

Much of the content of this draft guidance was incorporated into the two new final guidance documents released by the FDA in November 2017, which provide clarification on the same surgical procedure exemption (FDA, 2017n) as well as minimal manipulation and homologous use (FDA, 2017m) with specific examples as to how they relate to adipose tissue‐based products. Most unregulated stem cell clinics peddling adipose‐derived stem cell products tend to implement what is known as the stromal vascular fraction (SVF) in their products (Taylor‐Weiner & Graff Zivin, 2015). In order to prepare SVF products, these stem cells are isolated from the fat tissue within the liposuction aspirate in a manner that this draft guidance and the final guidance documents specially consider more than minimal manipulation and also incompatible with the same surgical exception (FDA, 2017n). Further, many clinics advertise applications of SVF for a variety of conditions and diseases that extend well beyond any homologous functions of adipose tissue as defined in this draft guidance and the final guidance on homologous use.

Even before the topics discussed in these draft guidance documents were finalized, the FDA was aware of the danger the existence of unregulated stem cell clinics and their unapproved products can present to unaware patients. In August 2017, the FDA took action against two stem cell clinics for “marketing stem cell products without FDA approval and significant deviations from current good manufacturing practice requirement” (FDA, 2017i). The first, in Florida, was administering SVF for a variety of conditions, including Parkinson's, heart, and pulmonary diseases (FDA, 2017o). The second clinic, in California, used live Vaccinia virus vaccine to create a stem cell product for cancer patients (FDA, 2017h). Although these actions mark an acceleration in FDA intervention, there remains a clear need for the FDA to enforce a firm stance on how adipose‐derived cell therapies are regulated.

### 2.4 Homologous Use of Human Cells, Tissues, and Cellular and Tissue‐Based Products (FDA, 2015a)

Although adipose‐derived cells are the most marketed cell type in unapproved stem cell clinics, this cell type only composes roughly 40–45% of available therapies and is closely followed by bone marrow‐derived cell therapies, which compose about 30–35% of a market share (Turner & Knoepfler, 2016), meaning that creating stiffer regulations only for adipose‐derived cell types will not regulate the entire market of unapproved clinics. Further, the range in diseases that unapproved stem cell clinics claim to treat range from orthopaedic injuries to neurological disorders, sexual enhancement procedures to sleep disorders, most of which lie outside what might be considered homologous uses of most commonly implemented cell types: adipose‐, bone marrow‐, or amniotic‐derived cells (Turner & Knoepfler, 2016). This draft guidance attempts to more specifically outline what qualifies as homologous use.

The draft guidance begins by repeating the original definition of “homologous use”, provided in 21 CFR Part 1271, as the “repair, reconstruction, replacement, or supplementation of a recipient's cells or tissues with an HCT/P that performs the same basic function or functions in the recipient as in the donor” (FDA, 2017d) and includes the new caveat that this definition must hold even “when such cells or tissues are for autologous use” (FDA, 2015a). The guidance further clarifies that the recipient cells or tissues do not necessarily need to be the same as those of the donor, nor must they perform all of the same functions, but they should perform at least “one or more of the same basic functions in the recipient as the cells or tissues in the donor” (FDA, 2015a). To determine if an HCT/P is intended for homologous use, the FDA examines the product labelling, marketing, and any other statements released by the manufacturer.

The draft guidance goes on to provide full definitions and examples of what qualifies as “repair, reconstruction, replacement, or supplementation” (FDA, 2015a). “Repair” was defined as “the physical or mechanical restoration of tissues, including by covering and protecting,” such as a skin graft treatment for a burn wound (FDA, 2015a). “Reconstruction” was considered to mean “surgical reassembling or re‐forming” of a tissue (FDA, 2015a). “Replacement” was defined as “substitution of a missing tissue or cell,” such as a cornea transplant (FDA, 2015a). The definition of “supplementation” was “to add to, or to complete,” such as implantation of bone chips to strengthen a bone defect (FDA, 2015a).

In this draft guidance, similar to the draft guidance on minimal manipulation, the FDA divides and further specifies the definition of homologous use for both structural and cellular/nonstructural tissues and provides a variety of relevant examples. In structural tissues, to qualify as homologous use, the HCT/P must serve a structural function, such as to “physically support or serve as a barrier or conduit, or connect, cover, or cushion” (FDA, 2015a). For example, a corneal graft to treat a blind patient would qualify as a structural homologous use; however, an amniotic membrane replacing bone tissue would be a nonhomologous use. Cellular or nonstructural tissue homologous uses would “generally be a metabolic or biochemical function in the recipient, such as hematopoietic, immune, and endocrine functions” (FDA, 2015a). Transplantation of haematopoietic stem/progenitor cells to reconstitute the haematopoietic system would qualify as a homologous use; however, haematopoietic stem/progenitor cell infusion into the heart to prevent tissue remodelling after a heart attack would be nonhomologous use. Both structural and cellular/nonstructural HCT/P can perform homologous uses even if they are acting in locations within the recipient's body that differ from where they act in the donor. For example, implanting a dermal graft to cover and protect a tendon would be a homologous use of the dermis; however, use of the same dermal product to replace or repair a tendon would be nonhomologous uses.

In a recent profile on unapproved cell therapy clinics, some businesses were found to advertise their stem cell therapies as treatments for over 30 diseases, most of which could not possibly qualify as homologous use under the definitions outlined in this draft guidance (Turner & Knoepfler, 2016). Concerningly, the sixth most common cell type marketed by these clinics was categorized “undefined” in this analysis (Turner & Knoepfler, 2016), which would pose complications given that the FDA specifies homologous use of products is determined by product “labeling, advertising, or other indications of manufacturer's objective intent” (Munos, 2009). Further, some clinics would interchange terms such as “placental stem cells” and “amniotic stem cells,” making the true source of such clinics' cells unclear (Turner & Knoepfler, 2016).

The content of this draft guidance was finalized in the recent guidance document on minimal manipulation and homologous use (FDA, 2017n). The recent final guidance explicitly highlights that any HCT/P used “for a myriad of diseases or conditions” is unlikely to be implemented homogenously (FDA, 2017n). Given this context and the recent legislation in Texas legalizing patient access to unapproved cell therapies as long as they are physician‐overseen (Servick, 2017), there is an apparent discrepancy between the federal regulatory policy and some states' policies in enforcing how cell therapies progress and are regulated within this rapidly growing, unregulated industry. The evolution and potential future judicial resolution of this seeming discrepancy in jurisdiction will be interesting to observe.

## 3 OVERVIEW OF THE BACKGROUND REPORT, “ADVANCING REGENERATIVE CELLULAR THERAPY: MEDICAL INNOVATION FOR HEALTHIER AMERICANS,” THE REPORT INSPIRING THE REGROW ACT

In response to the lack of updated federal legislation on HCT/Ps, in December 20, 2015, the BPC think tank released “Advancing Regenerative Cellular Therapy: Medical Innovation for Healthier Americans,” a report that outlines the current status of adult cell therapies and their current barriers to clinical translation due to outdated regulations (BPC, 2015). The report begins by outlining what adult cell therapies are, providing a cursory overview of the extensive regenerative adult cell therapy clinical trial history, and describing the slow progress that has been made by the FDA to create appropriate regulatory practices for cell‐based therapies (BPC, 2015). As part of the argument the report builds for changing the legislative policies surrounding HCT/P regulation, the BPC gives a rough overview of the literature surrounding cell therapy trials and notes that the vast body of studies seem to affirm the overall safety of adult cell therapy trials (BPC, 2015).

**Figure 1:**
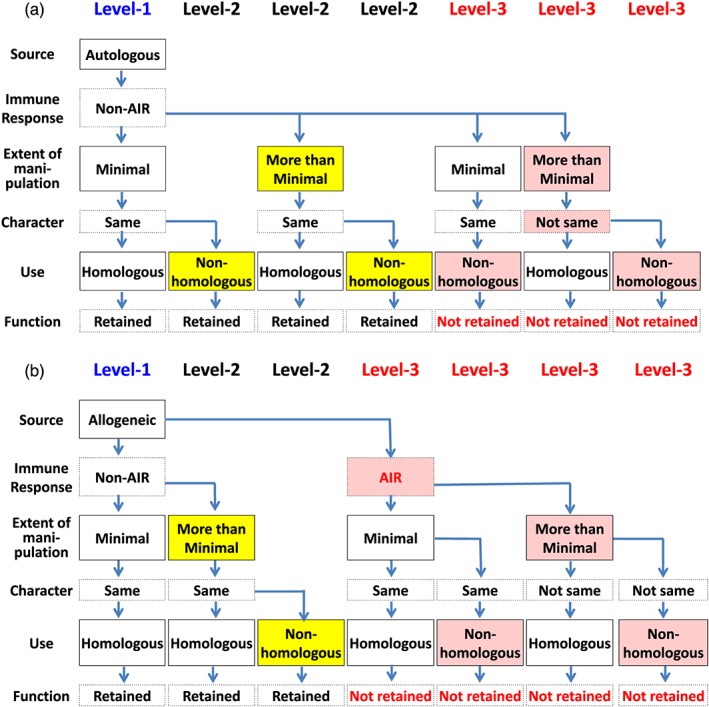
Flowchart to decide categorizations of autologous and allogeneic Human Cells, Tissues, and Cellular and Tissue‐based Products as described by the BPC Report. AIR, induce an adverse immune response; Non‐AIR, do not induce any adverse immune response. Yellow‐coloured boxes indicate new key characteristics of Human Cells, Tissues, and Cellular and Tissue‐based Products (HCT/Ps) under BPC Report Level 2. Reddish‐coloured boxes indicate new key factors of HCT/Ps under BPC Report Level 3. These figures outline the HCT/P categorizations recommended in the Bipartisan Policy Center released in the report titled “Advancing Regenerative Cellular Therapy: Medical Innovation for Healthier Americans.” The figures organize how autologous or allogeneic HCT/Ps would be distributed into Level 1, 2, or 3. HCT/Ps are organized according to source: autologous (a) or allogeneic (b), elicited immune response (AIR or non‐AIR), extent of manipulation (minimal manipulation or more than minimal manipulation), cell character and function after manipulation (retaining the same character and function or not retaining the same character and function), and cell use (homologous use or no homologous use) [Colour figure can be viewed at http://wileyonlinelibrary.com]

At the end of the report, the BPC includes a summary of proposed policy changes outlined in a modified hierarchy of the three tiers created by the FDA in 2001 (BPC, 2015). The BPC renames the three tiers Levels 1, 2, and 3 and includes categorization criteria according to any adverse immune response, which impacts their safety or efficacy (Figure 1; BPC, 2015). Notably, the proposed regulation requirements for Level 1 (described by the FDA as Tier 1, the lowest risk tier) would continue to be regarded as standard medical practice and therefore exempt from FDA preapproval (BPC, 2015). The types of cell‐based therapies qualifying for Level 1 regulation were more specifically outlined and broadened to include autologous cells as well as allogeneic bone marrow and cord blood cells that are minimally manipulated and implemented homologously (previously, such HCT/Ps would have fallen under Tier 2 as 361 products in the FDA hierarchy; BPC, 2015). Similarly, the regulation requirements for Level 3 (described by the FDA as 351 products in Tier 3, the highest risk tier) would remain the same as in the original hierarchy outlined by the FDA and therefore subject to the standard pathway regulations, including a required investigational new drug application and full BLA (BPC, 2015). The types of cell‐based therapies that would fall into Level 3 were more explicitly laid out in the report and were described to include allogeneic cells that cause an adverse immune response or autologous cells that do not maintain their character/function or do not help restore function (BPC, 2015). The most substantive revision of the three tiers was in the creation of a new pathway within Level 2 for autologous and allogeneic cell‐based therapies that do not induce an adverse immune response, a categorization that previously would have fallen into the third tier of the FDA hierarchy (BPC, 2015). These cells could be more than minimally manipulated but should maintain their character and functionality (BPC, 2015). They could also be implemented for nonhomologous uses but should still improve function (BPC, 2015). This pathway would still require an investigational new drug; however, there would be no required Phase III trials, and only a preliminary indication of safety and efficacy would be necessary to obtain a time‐limited, conditional approval by the FDA (BPC, 2015). Patients would gain limited access to the conditionally approved therapies, but the FDA would require sufficient monitoring and reporting of results. A BLA, based on the data collected over 3 years, or across a subsequently negotiated, longer time frame, would be required for the product to remain on the market (BPC, 2015). Other proposed changes included an expedited linked approval process for cell therapies used in conjunction with medical devices. Figure 1 show detailed information on this new regulatory framework for cellular therapy proposed by the BPC (BPC, 2015).

The key principle driving the proposed BPC's policy was to promote the progress of cellular therapies in the United States that are necessary for improving public health care options and promoting domestic innovation in the regenerative medicine sphere (BPC, 2015). The BPC‐recommended pathway was designed to facilitate submission of clinical data to the FDA and streamline approval of a subset of cellular therapies demonstrating safety and effectiveness that is presently impossible under current regulation (BPC, 2015). This report ended up serving as the framework from which the REGROW Act was drafted by the U.S. Congress in 2016.

## 4 OUTLINE OF THE PROPOSED REGROW ACT

On March 16, 2016, U.S. Senators Mark Kirk, Joe Manchin, and Susan Collin and U.S. Representatives Mike Coffman, Mark Takai, and H. Morgan Griffith introduced the REGROW Act of 2016 (S. 2689/H.R. 4762; Coffman, Takai, & Griffith, 2016; Kirk, Manchin, & Collon, 2016), a bill designed to facilitate faster adoption of adult human cellular therapies. This Act was not enacted during the 114th Congress and therefore died when the Congress ended it at the start of January 2017 (GovTrack US, 2017). However, the concept of adapting an accelerated approval pathway for select eligible cell therapies was incorporated into the 21st Century Cures Act, which was signed into law on December 13, 2016, by former President Obama (GovTrack US, 2017).

Table 2 shows detailed information on the contents of the proposed REGROW Act (Coffman et al., 2016; Kirk et al., 2016). The most substantial changes proposed within the bill were motivated by the changes recommended in the BPC Report and were related to the approval process for cellular therapies and the classification of cellular therapeutics (BPC, 2015). The Act proposed the creation of a 5‐year conditional use period for cellular or tissue therapeutics demonstrating sufficient safety and efficacy, without Phase III investigation (Coffman et al., 2016; Kirk et al., 2016). To be approved under this proposed conditional approval system, the eligible products are adult human cells and tissues that have been either minimally manipulated for nonhomologous use or more than minimally manipulated products for homologous or nonhomologous use but “do not provoke a significant unintended immune response in the recipient” (Coffman et al., 2016; Kirk et al., 2016). The adult cells and tissue must be used for “a specific indication” and should “achieve or restore, the same, or similar, function in the recipient as the donor” (Coffman et al., 2016; Kirk et al., 2016). Within 5 years of conditional approval, an annual report and adverse event reports would need to be submitted to the FDA before and in addition to “an application for approval of a biological product” under the new legislation (Coffman et al., 2016; Kirk et al., 2016). During the conditional approval period, doctors must inform each patient of the products' conditional approval status.

**Table 2:** Proposed REGROW Act

Section 1. Short title
Reliable and Effective Growth for Regenerative Health Options that Improve Wellness (REGROW Act)
Section 2. Cellular therapeutics
Current pathways—No part of the proposed bill is intended to modify the current pathway to market is overseen by the Food and Drug Administration (FDA), governed through Sections 351 and 361 of the Public Health Service (PHS) Act.
Approval for therapies—Section 351 of the PHS Act is amended by adding the following:
Sec. 351B. Approval for cellular therapies
Conditional approval of cellular or tissue therapeutics: Calls for creation of a program for the conditional approval of safe cellular therapeutic products without Phase III clinical trials.
Additional requirements for conditional approval: Qualified conditionally approved products can enter a 5‐year conditional use period if the following requirements are met:
Are “adult human cells or tissues”
Examination of immunogenicity reveals no “significant unintended immune response”
Are (A) “minimally manipulated for a non‐homologous use” or (B) “more‐than‐minimally manipulated for a homologous or non‐homologous use, but are not genetically modified”
Are “produced for a specific indication”
Perform “the same, or similar, function in the recipient as in the donor”
A biological product approval application (described under PHS Act Section 351(a)) is submitted within 5 years
“Annual reports and adverse event reports” should be submitted throughout the conditional approval period until the biological product
Approval application is approved
Submitted a sponsor application to treat patients within the 5‐year conditional use period
The product has not been previously conditionally approved for the same use
Informed use: Patients must be notified of the conditional approval status of the product and that it has not been proven efficacious.
Stem cell banking: “Public and private cord blood banks, tissue banks, and bone marrow repositories shall be in full compliance with good tissue practice requirements” under 21 CFR 1271.
Section 3. Devices used in recovery, processing, and delivery of cellular therapeutics
Clearance—Outlines editing changes to be made to Section 510(k) of the Federal Food, Drug, and Cosmetic Act (FFDC Act).
Clearance or approval of cellular therapeutics.
Sec. 515B. Classification of cellular therapeutics
Cellular therapeutic device clearance or approval will depend on in vitro testing performance.
Classifications of devices “used for cell therapy,” should be determined by their general uses: “harvesting, delivery, or processing cells and sustaining the viability and function of the cells *in vivo*.”
No additional clearance required for approved devices to be used with cells unless they impact the intended use of the device.
Reclassification of low risk class III products and those lacking previous approval without cells are governed by the FFDC Act.
Combination products—Outlines editing changes to be made to Section 503 of the FFDC Act.
Section 4. Guidance: Amended regulations
Guidance—A draft guidance may be released to clarify any of the presented changes within 1 year of enactment, and a final guidance will be issued within 180 days from the end of the comment period.
Amended regulation.
In general—Any amendments to 21 CFR to clarify the presented changes should be completed within 1 year of the enactment.
Procedure—Outline of amendment process.
Public meeting—Within 90 days of enactment, at least one public meeting will be held to discuss the regulation of HCT/Ps.
Section 5. Regenerative medicine standards—Consultation of relevant stakeholders to develop transparent standards for regenerative medicine products

*Note*. The REGROW Act was proposed at 114th Congress 2D Session as S. 2689 and H.R. 4762 to amend the Federal Food, Drug, and Cosmetic Act with respect to cellular therapies by Senators Mark Kirk, Joe Manchin, and Susan Collins and Representatives Mike Coffman, Mark Takai, and Morgan Griffith. 21 CFR1271 = Part 1271 of Title 21, Code of Federal Regulation; FFDC = Federal Food, Drug, and Cosmetic Act; HCT/Ps = Human Cells, Tissues, and Cellular and Tissue‐based Products; PHS = Public Health Service.

The expedited approval pathway presented in the REGROW Act would have not only accelerated approval for a broader group of eligible regenerative medicine therapeutics, rather than just for those addressing serious or life‐threatening illnesses, as under the existing guidelines, it also would have enabled these products to enter the market before traditional Phase III testing. Critics of this proposed legislation urged that the appropriate existing expedited pathways already existed and warned that circumventing Phase III trials would expose patients to ineffective and potentially harmful products (Turner & Knoepfler, 2016). The attrition rate for prospective drugs and therapeutics can be as high as 40% in Phase III testing due to concerns over safety or inefficacy (Servick, 2017). Further, Phase III trials often provide large‐scale patient data comparing the current standard of care with the experimental therapy and can provide physicians with important data for instructing and recommending patient use. Critics argued that the financial burden for covering the equivalent of Phase III trial costs for potentially ineffective treatments would be redistributed from commercial entities to the government, patients, private health care, and insurance companies (Editorial, 2016).

Supporters of the REGROW Act insisted that the traditional clinical trial process was too arduous, time‐consuming, costly, and likely prevented perfectly safe and effective treatments from reaching patients in critical need (BPC, 2015). The amount of time it takes to get potential drugs and therapies from patenting to commercialization has increased over the years with average estimates now well over a decade (Pammolli, Magazzini, & Riccaboni, 2011). The costs of taking a product through the entire development process has also exponentially increased over the past decades, and now total costs are approximately $2 billion to take a product to the market (Munos, 2009). Supporters of the REGROW Act also pointed to the recent changes in British and Japanese legislation that may enable these countries to break ahead or attract previously U.S.‐based companies abroad to drive the progress of regenerative medicine products (RMPs) and therapies under more amenable legislative conditions (BPC, 2015). Further, supporters viewed the changes proposed by this Act as an essential pathway to treatment access for patients suffering from rare diseases that could be treated with RMPs and that currently slip through the existing expedited regulations or may not yield the required patient numbers for conducting timely Phase III clinical trials (Cetrulo, 2016).

The 21st Century Cures Act, enacted in December 2016 (Public Law 114‐255 114th Congress—Dec. 13, 2016), is seen by many as the replacement legislation to the REGROW Act as it includes provisions to ease the approval requirements for “regenerative medicine advanced therapy (RMAT)” category for drugs (FDA, 2017l) that encompass (a) “regenerative medicine therapies, including cell therapies, therapeutic tissue engineering products, human cell and tissue products, and combination products,” aside from those that are regulated as 361 products; (b) drugs that “treat, modify, reverse, or cure a serious or life‐threatening disease or condition”; or (c) “preliminary clinical evidence indicates that the drug has the potential to address unmet medical needs for such a disease or condition” (Public Law 114‐255 114th Congress—Dec. 13, 2016). This new RMAT designation targets many products that would have been previously regulated as 351 products. Qualifying therapies would be eligible, through consultation with the FDA, for custom‐designed, expedited pathways where products could undergo a “priority review” or where the alternative intermediate endpoints may be substituted (Public Law 114‐255 114th Congress—Dec. 13, 2016). RMAT therapies may still be subject to “postapproval requirements” where subsequent clinical data must be submitted or “postapproval monitoring” must be conducted (Public Law 114‐255 114th Congress—Dec. 13, 2016). The bill also called for the FDA to work with the National Institute of Standards and Technology to create standards for evaluating “regenerative medicine and advanced therapies” (FDA, 2017e). Provisions encouraged collection of “patient experience data” to be considered alongside clinical data as part of the process of drug development and review. This legislation, however, maintains the standard of evidence required for therapeutic approval and does not limit the FDA's authority in the approval of these therapies.

## 5 SUMMARY OF THE FDA‐APPROVED PRODUCTS REGULATED UNDER SECTION 351 OF THE PHS ACT IN THE UNITED STATES

As of the end of December 2016, 24 cell‐based products and related products regulated under Section 351 of the PHS Act and 21 CFR Part 1271 had been approved in the United States by the FDA (Table 3; FDA, 1997a, 1997b, 1998, 2001a, 2001b, 2001d, 2007, 2010, 2011a, 2011b, 2012a, 2012b, 2012c, 2012d, 2013a, 2013b, 2015b, 2016a, 2016b, 2016e, 2016f, 2017a, 2017f, 2017j). Before 2005, most products were aimed at wound healing applications (Yano et al., 2015; Yano, Tsuyuki, Watanabe, Kasanuki, & Yamato, 2013). After 2005, the products now target a broader range of applications, including some of the most life‐threatening diseases, such as heart disease or cancer. The low number of these approved 351 products is reflective of the lengthy and costly regulation that these HCT/Ps were forced to follow in order to obtain FDA approval.

**Table 3:** Summary of the Food and Drug Administration approval of products regulated under Section 351 of the Public Health Service Act in the United States

Generic name (trade name)	Approval date (approval pathway)	Marketing authorization holder	Intended target disease/condition
**Autologous**			
Autologous cultured chondrocytes (Carticel™)	August 22, 1997 (BLA), September 26, 2017	Vericel Corporation, Cambridge, MA	Symptomatic cartilage defects; Discontinued to being marketed
Cultured epidermal autografts (Epicel®)	October 25, 2007 (HDE); February 18, 2016	Vericel Corporation, Cambridge, MA	Deep dermal or full thickness burns; Pediatric use
Sipuleucel‐T (PROVENGE®)	April 29, 2010 (BLA)	Dendreon Co., Seattle, WA	Asymptomatic or minimally symptomatic metastatic castrate resistant (hormone refractory) prostate cancer
Azficel‐T (Laviv®)	June 21, 2011 (BLA)	Fibrocell Science Inc., Boulder, CO	Moderate to severe nasolabial fold wrinkles
Autologous cultured chondrocytes on a porcine collagen membrane (MACI)	December 13, 2016 (BLA)	Vericel Corporation, Cambridge, MA	Full‐thickness knee cartilage defects
Tisagenlecleucel (KYMRIAH)	August 30, 2017 (BLA); May 1, 2018 (BLA)	Novartis Pharmaceuticals Corporation, East Hanover, NJ	Patients up to 25 years of age with B cell precursor acute lymphoblastic leukemia (ALL; cell‐based gene therapy); Adult patients with relapsed or refractory (r/r) large B‐cell lymphoma
Axicabtagene ciloleucel (YESCARTA)	October 18, 2017 (BLA)	Kite Pharma, Incorporated, Santa Monica, CA	Adult patients with large B‐cell lymphoma (cell‐based gene therapy)
**Allogeneic**			
Interactive wound and burn dressing (formerly, Dermagraft‐TC™; currently, TransCyte®)	March 18, 1997 (PMA)	Organogenesis, Inc., Canton, MA	Surgically excised full‐thickness and deep partial‐thickness thermal burns
	August 14, 1998 (PMA supplement)		
			Mid‐dermal to indeterminate‐depth burn wounds
Living Skin Equivalent (LSE) Graftskin (Apligraf™)	May 22, 1998 (PMA)	Organogenesis Inc., Canton, MA	Noninfected partial and full‐thickness skin ulcers
	June 20, 2000 (PMA supplement)		
			Diabetic foot ulcers
Interactive wound and burn dressing (Composite Cultured Skin)	February 21, 2001 (HDE)	Ortec International, Inc., New York City, NY	Mitten hand deformity
Interactive wound and burn dressing (Orcel™)	August 31, 2001 (PMA)	Ortec International, Inc., New York City, NY	Burn wounds
Interactive wound dressing (Dermagraft®)	September 28, 2001 (PMA)	Advanced BioHealing, La Jolla, CA (2001–2002)	Full‐thickness diabetic foot ulcers
		Smith & Nephew, La Jolla, CA (2002–2006)	
		Advanced BioHealing, La Jolla, CA (2006–2011)	
		Shire Regenerative Medicine, San Diego, CA (2011–) Organogenesis Inc., Canton MA	
Allogeneic cultured keratinocyte and fibroblast in bovine collagen (Gintuit)	March 9, 2012 (BLA); September 3, 2014	Organogenesis Inc., Canton, MA	Mucogingival conditions; Discontinued to being marketed
BCG Live (Intravesical) (TheraCys®)	November 8, 2012 (BLA); November 2016	Sanofi Pasteur Limited, West Toronto, Ontario, Canada	Non‐muscle invasive bladder cancer; Discontinued to being marketed
Talimogene laherparepvec (IMLYGIC)	October 27, 2015 (BLA)	Amgen, Inc., Thousand Oaks, CA	Unresectable cutaneous, subcutaneous, and nodal recurrent melanoma lesions (genetically modified live oncolytic herpes virus therapy)
Voretigene neparvovec‐rzyl (LUXTURNA)	December 19, 2017 (BLA)	Spark Therapeutics, Inc., Philadelphia, PA	Confirmed biallelic *RPE65* mutation‐associated retinal dystrophy (gene therapy product)
**Allogeneic (unrelated allogeneic placental/umbilical cord blood products and related products)**			
HEMACORD; HPC, Cord Blood (Hemacord™)	November 10, 2011 (BLA)	New York Blood Center, Inc., New York, NY	Haematopoietic system disorders
HPC, Cord Blood (None)	May 24, 2012 (BLA)	Clinimmune Labs, University of Colorado Cord Blood Bank, Aurora, CO	Haematopoietic system disorders
HPC, Cord Blood (Ducord)	October 4, 2012 (BLA)	Duke University School of Medicine, Translation Cell Therapy Center, Carolinas Cord Blood Bank, Durham, NC	Haematopoietic system disorders
HPC, Cord Blood (ALLOCORD)	May 30, 2013 (BLA)	SSM Cardinal Glennon Children's Medical Center, St. Louis, MO	Haematopoietic system disorders
HPC, Cord Blood BLA 12543 (None)	June 13, 2013 (BLA)	LifeSouth Community Blood Centers, Inc., Gainesville, FL	Haematopoietic system disorders
HPC, Cord Blood (None)	January 28, 2016 (BLA)	Bloodworks, Seattle, WA	Haematopoietic system disorders
HPC, Cord Blood (Clevecord)	September 1, 2016 (BLA)	Cleveland Cord Blood Center, Warrensville Heights, OH	Hematopoietic system disorders
Sterile cord blood collection unit with anticoagulant citrate phosphate dextrose solution USP (CPD) (None)	December 21, 2016 (NDA)	Maco Productions S.A.S., Duluth, GA	Umbilical cord blood collection (40–250 ml)

*Note*. BLA = Biologics License Application; HDE = Humanitarian Device Exemption; PMA = PreMarket Approval Application; BCG = Bacillus Calmette–Guérin; HPC = Haematopoietic Progenitor Cells; CPD = Citrate Phosphate Dextrose; USP = United States Pharmacopoeia; NDA = New Drug Application.

## 6 DISCUSSION

HCT/Ps that are subject to FDA regulation as 351 products have a costly and lengthy process to market, requiring a full premarket BLA and the same premarket and postmarket regulation as medical devices, drugs, or biologics. HCT/Ps that qualify as standard medical practice or 361 products can circumvent this lengthy regulatory process as they are not subject to FDA preapproval or regulation. The boundary that defines 351 and 361 products has been the subject of much recent regulatory debate. The FDA released the four draft guidance documents outlined in this article to further define and, in some instances, narrow criteria that would enable an HCT/P to qualify as a 361 product. In 2017, two final guidance documents were released by the FDA that finalized many of the topics presented in these four draft guidance documents.

The first finalized guidance document, “Same Surgical Procedure Exception Under § 1271.15(b): Questions and Answers Regarding the Scope of the Exception,” finalizes the limited scope of the same surgical exception draft guidance, covering only the autologous implementation of HCT/Ps in procedures conducted at the same establishment. It further clarifies that HCT/Ps that qualify for this exemption are not considered 361 products but qualify as first tier, lowest risk products and are considered current standard medical practice and therefore not subject to FDA regulation (FDA, 2017n). The second final guidance called “Regulatory Considerations for Human Cells, Tissues, and Cellular and Tissue‐Based Products: Minimal Manipulation and Homologous Use” defines and provides specific examples of procedures that qualify as minimal manipulation and applications that qualify as homologous use, many further clarifying descriptions originally described in the homologous use and minimal manipulation draft guidance documents (FDA, 2017m). Interestingly, these new guidance documents explain that they replace the draft guidance document on adipose tissue with specific examples explicitly stating that SVF products cannot qualify for the same surgical exemption, their isolation procedure does not qualify as minimal manipulation, and their application cannot be considered homologous as they cannot perform the structural or supportive role of the original adipose tissue (FDA, 2017m). The FDA is allowing the manufacturers 3 years to come into compliance with these more restrictive guidance documents, however is currently targeting some of the worst clinics.

The original release of the four draft guidance documents outlined in this article marked the start of the FDA's aims to enforce its jurisdiction and narrow the definition of 361 products. In response to these original draft guidance documents, controversial proposed changes were presented in the BPC background report “Advancing Regenerative Cellular Therapy: Medical Innovation for Healthier Americans” (BPC, 2015) and the REGROW Act (Coffman et al., 2016; Kirk et al., 2016). These changes attempted to counteract FDA action by reducing the number of products that would fall under the FDA jurisdiction through redefinition of the product regulatory categorizations and reduction of necessary clinical trial evidence before product release onto the market. Many of these modifications resembled the conditional and time‐limited authorization for RMPs in Japan. The concept of creating an expedited approval pathway for certain therapies was not new. “Accelerated approval” pathways for drugs and biologics for serious or life‐threatening illnesses already exist in the United States (Yano, Watanabe, & Yamato, 2016). “Conditional market authorization” of drugs for serious and life‐threatening diseases, emergency situations, and orphan diseases as well as “market authorization under exceptional circumstance” of drugs also exist in the European Union (Yano et al., 2016). In 2014, Japan became the first country to introduce an expedited approval system that would create a conditional approval pathway for RMPs in the “Pharmaceuticals, Medical Devices and Other Therapeutic Products (PMDA) Act” (Hara, Sato, & Sahara, 2014). Under the new Japanese system, RMPs, regardless of whether they treat life‐threatening diseases, can enter the market after confirming safety and demonstrating efficacy. However, clinical efficacy in large‐scale clinical trials must be demonstrated and submitted to the relevant authorities after 7 years. This Japanese legislation was the initial spark for much of this controversial discussion about regulation of cell therapies amongst the international scientific community (Hara et al., 2014; Konomi, Tobita, Kimura, & Sato, 2015). Critics of the newly implemented conditional and time‐limited authorization system for RMPs in Japan raised concerns that these changes may be too lax and may “flood” the Japanese market with ineffective therapies (Editorial, 2015). Although it will take years before the impact of the recent changes in the Japanese system are fully understood, the first RMP, HeartSheet®, was already approved by the Japanese regulatory authority (Ministry of Health, Labour and Welfare) on September 18, 2015, under the new act (Pharmaceuticals and Medical Devices Agency, 2015). As the clinical aim of HeartSheet® is the treatment of heart disease, it already targets a class of regenerative therapy beyond the scope of the 23 products (Table 3) that have been approved in the United States.

Although the 21st Century Cures Act creates a regulatory exception for a selection of previous 351 HCT/P therapies, it does not remove the need for FDA regulation over the approval process. In response to the 21st Century Cures Act, the FDA released a draft guidance document outlining the alternative approval pathways available to RMATs (FDA, 2017g). The flexible new system accepts surrogate endpoints and the ability to consult with the FDA earlier in the approval process to establish case‐by‐case criteria, making the pathway to approval potentially faster and more sensitive to the diverse range of candidate therapies. However, these case‐by‐case considerations make the pathway to approval vaguer overall and still maintain, if not strengthen, FDA oversight over the entire process. Further, despite these changes, to date, there are still some HCT/Ps that cannot qualify for this RMAT designation and will remain regulated as 351 products. This recent further strengthening of the FDA's control as the final gatekeeper of therapeutic approval grew out of four draft guidance documents that were released by the Agency between 2014 and 2015 and has been fuelled in response to ideas contained within the REGROW Act and recent deregulatory actions. The vague case‐by‐case regulatory pathway laid out by the FDA's draft guidance on RMAT alternative expedited approval pathways will make it challenging to gauge how the FDA's perspective on RMP development and approval will change in the future. Further, unlike the standardized safety criteria laid out for evaluating cell therapy safety and risk in the BPC Report, the case‐by‐case evaluation will obscure the evaluation process from anyone outside of the FDA, making it difficult to ensure that evaluation is consistent across candidate therapies.

Under the previous U.S. regulations, it would be hard to imagine applications of cell‐based therapies that would fulfil the requirements for minimal manipulation and homologous use in such serious diseases as heart disease, seemingly condemning products seeking FDA approval to a longer, more expensive regulatory pathway. Thus it is not surprising that the United States was considered to have the highest density of “stem cell tourism” clinics in the world in 2014 (Taylor‐Weiner & Graff Zivin, 2015). The regulatory environment in the United States fostered opposing extremes: products forced into a costly regulatory labyrinth with little hope of ever making it to market, or “experimental” unregulated products circumventing the entire system exploiting the free market and desperate patients' hopes for relief, with little productive middle ground in between that would safely provide patients in need with safe, timely treatments for a variety of life‐threatening diseases (Taylor‐Weiner & Graff Zivin, 2015). The recent legislative action introducing the new RMAT designation and the supplemental FDA draft guidance guidelines may reduce controversial stem cell tourism, by providing an alternative pathway of products that target serious conditions, unmet medical needs, and/or rare diseases. The FDA's finalization of the two draft guidance documents on the same surgical exemption as well as minimal manipulation and homologous use also define narrower guidelines, reducing the ability for suppliers to skirt FDA regulation. The final guidance on homologous use and minimal manipulation explicitly states that HCT/Ps implemented “for a myriad of diseases or conditions” are unlikely compatible with the criteria of homologous use required for HCT/Ps to qualify as 361 products. This guidance also lays out a warning that products presenting a higher risk based on their “site of administration” and those implemented for “non‐homologous use, particularly those intended to be used for the prevention or treatment of serious and/or life‐threatening diseases and conditions” will be the first to be targeted by FDA compliance enforcement. With exception of the most nefarious clinics, which are currently FDA scrutiny, manufactures have been given 3 years to come into compliance, but this premeditated description of products that will be subject to regulation make the FDA's intentions clear, and only time will tell if the Agency has the necessary infrastructure to broadly enforce compliance.

The recent action by the FDA warning several cell therapy clinics that they need to acquire FDA approval in order to sell some of their products shows that the Agency certainly has an interest in attempting to enforce its authority.

The original draft guidance on adipose tissue‐derived products highlighted that implementation of adipose tissue‐derived HCT/Ps for breast augmentation would not be considered a homologous use. However, in the final guidance, this implementation is listed as a homologous use, as cushioning and support are considered some of the original functions of the harvested adipose tissue. Although a seemingly subtle difference, the original draft guidance could, arguably, have been seen as FDA encroachment on medical practice. The regulation of medical practice is outside of the jurisdiction of the FDA as it can only control the dissemination and regulation of medical products. Under U.S. law, the authority to regulate the practice of medicine lies with the individual states. The FDA, however, has been extending its authority and in some instances, arguably, has encroached on the practice of medicine related to HCT/P therapies. The revision of the categorization of some adipose tissue‐derived products for breast augmentation in the final guidance may reflect the FDA's cognizance of this boundary to its authority. However, considered in the context of the “Right to Try” movement and the deregulation of stem cell therapies in Texas, this overlap of authority may foreshadow future clashes over jurisdiction. The power balance between the U.S. federal and state governments is complex, and ultimate changes to this distribution of power can only the settled through the slow justice of the judicial branch.

Although the REGROW Act died in the previous Congress (GovTrack US, 2017), some believe that a bill with similar more detailed regulation of the HCT/P therapeutic approval process can be anticipated to reappear in the future, as there is still much room for optimization in the translation of bench‐top discoveries to bedside therapies within the existing U.S. system. Through the recent release of several draft guidance documents (FDA, 2017b, 2017l) and warnings to stem cell clinics (FDA, 2017h, 2017i), the FDA has been taking steps to exert its authority. At the same time, in the wake of the REGROW Act (Coffman et al., 2016), the gathering momentum of the “Right to Try” movement (Goldwater Institute, 2017; Pear & Kaplanug, 2017; Servick, 2017), and the current deregulatory political environment in the United States, there appears to be legislative momentum that could begin to create legal avenues by which the FDA could be side‐stepped and the diplomatic provisions of the 21st Century Cures Act could become a thing of the past.

## CONFLICT OF INTEREST

K. Yano is also an employee of Medtronic Japan Co., Ltd. M. Yamato is a shareholder of CellSeed Inc.

## AUTHOR CONTRIBUTIONS

Conceptualization: K.Y. and M.Y. Writing, original draft: K.Y. and M.Y. Writing, review and editing: K.Y., A.S., and M.Y. Supervision: M.Y.
